# Targeting the tumor microenvironment to improve clinical outcomes in triple negative breast cancer patients and bridge the current disparity gap

**DOI:** 10.3389/fimmu.2024.1428118

**Published:** 2024-07-12

**Authors:** Malak Alharbi, Arya Mariam Roy, Jayasree Krishnan, Pawel Kalinski, Song Yao, Shipra Gandhi

**Affiliations:** ^1^ Department of Medicine, Roswell Park Comprehensive Cancer Center, Buffalo, NY, United States; ^2^ Department of Internal Medicine, King Abdulaziz University Hospital, Jeddah, Saudi Arabia; ^3^ Department of Immunology, Roswell Park Comprehensive Cancer Center, Buffalo, NY, United States; ^4^ Department of Cancer Prevention and Control, Roswell Park Comprehensive Cancer Center, Buffalo, NY, United States

**Keywords:** triple negative breast cancer, racial disparities, tumor microenvironment, exhausted CD8 + T cells, TLR3 agonist

## Abstract

Triple negative breast cancer (TNBC) is a heterogenous disease that disproportionately affects Black women. TNBC outcomes among Black women are dismal secondary to multiple factors, such as poor healthcare accessibility resulting in delays in diagnosis, and aggressive disease biology in addition to a pro-tumor immune microenvironment (TME). Black women with breast cancer exhibit elevated levels of serum pro-inflammatory cytokines, and a pro-tumorigenic TME with higher immunosuppressive regulatory T cells (Tregs), M2 macrophages and exhausted CD8^+^ T cells. We have shown that the combined use of toll-like receptor 3 (TLR3) ligands with interferon-α (chemokine modulation: CKM) is able to enrich the tumor with CD8^+^ T cells, while not increasing immunosuppressive cells. Recent clinical trials have revealed the efficacy of immune checkpoint inhibitors (ICI) in rejuvenizing exhausted CD8^+^ T cells. We hypothesize that strategies to modulate the TME by enriching chemokines that attract CD8^+^T cells followed by reversal of CD8^+^ T cell exhaustion (ICI), when added to standard treatment, could potentially improve clinical outcomes, and mitigate the racial disparities in TNBC outcomes between Black and White Women.

## Introduction

Triple negative breast cancer (TNBC) accounts for 15- 20% of all breast cancers (1), higher among Black population, with an age-adjusted incidence of 33.8 cases per 100,000, compared to 17.5 per 100,00 cases in White women (2). Additionally, TNBC in Black individuals carries a higher mortality rate of 29.2 out of 100,000 compared to 20.6 out of 100,000 in White women (3). Besides other factors associated with poor access to care, and delayed diagnosis; biological factors such as differences in tumor biology (4), tumor microenvironment (TME) and host immunity (5), contribute to racial disparities in clinical outcomes of TNBC; thus, there has been a recent interest in exploring the role of TME in breast cancer racial disparities. However, the under-representation of Black patients in clinical trials, including those with immunotherapy, has impeded the understanding of TNBC disparities, as evidenced by the lack of race data and race-specific clinical outcomes reported in KEYNOTE-522 (6) and GeparNuevo (7) and by the inclusion of only a few Black women (9%) in the pembrolizumab arm of I-SPY2 trial (8).

## Understanding differences in tumor microenvironment across breast cancer from different racial groups

### Tumor infiltrating lymphocytes and exhausted CD8^+^ T cells

**Figure 1:**
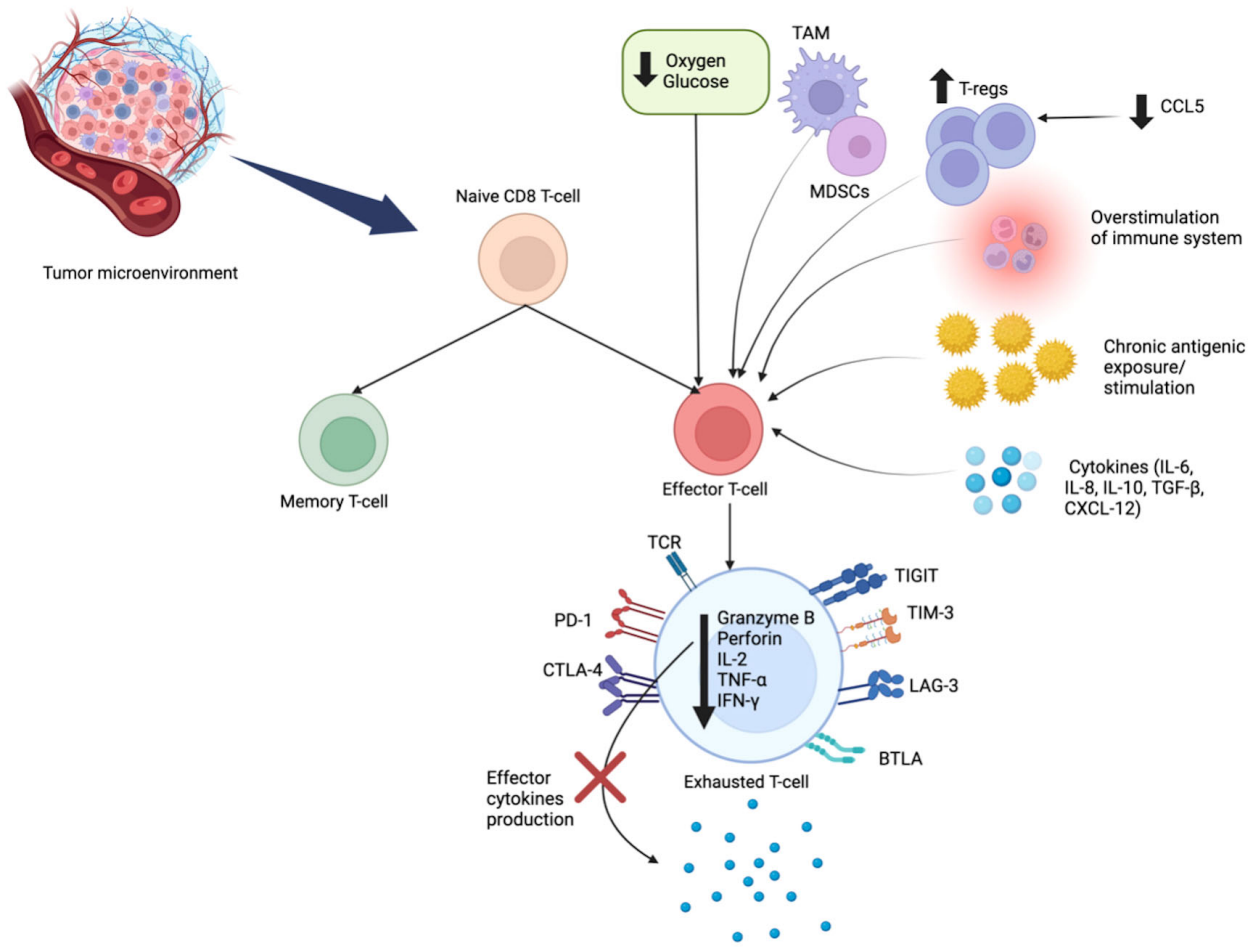
Mechanisms of CD8^+^ T Cell Exhaustion. CD8^+^ T cell exhaustion is a dynamic process, resulting from chronic antigenic overstimulation, hypoxia, and the immunosuppressive cells (TAM, MDSCs, Tregs) and cytokines (IL-10, TGF-β). In this state, effector cells progressively lose their cytotoxic ability. Exhausted CD8^+^ T cells have a unique metabolic pattern and epigenetic features, with sustained expression of inhibitory receptors: CTLA-4, PD-1, BTLA, LAG-3, TIM-3 and TIGIT. Exhausted T cells have decreased production of granzyme B, perforin, and effector cytokines (IL-2, TNF-α, IFN-γ). IL, interleukin; TNF-α, tumor necrosis factor alpha; IFN-γ, interferon gamma; Tregs, regulatory T cells; MDSCs, myeloid derived suppressor cells; TAM, tumor-associated macrophages; TGF-β, Transforming growth factor beta; CTLA-4, T lymphocyte antigen-4; PD-1, programmed cell death protein-1; LAG-3, lymphocyte activation gene 3 protein; BTLA, B and T-lymphocyte attenuator; TIM-3, T cell immunoglobulin domain and mucin domain protein 3; TIGIT, T cell immunoreceptor with Ig and ITIM domains.

TIL infiltration has been shown to be an independent prognostic marker in TNBC. Prior TNBC Phase *III* trials have shown that high stromal and intratumoral TIL numbers are associated with improved disease-free survival (DFS), distant recurrence-free survival (DRFS), and overall survival (OS) (9). A previous study from our group based on a cohort of 1315 patients (920 Black patients and 395 White patients) showed that tumors from Black patients expressed significantly higher pathological TIL score compared to White patients (P<0.001) (10). Although there is no racial difference in the fraction of CD8^+^ T cells, tumors from Black patients have higher expression of CD8^+^ T cell exhaustion markers (PD-1, LAG3) and eomesodermin (EOMES), a pivotal transcription factor upregulated in exhausted CD8^+^ T cells (11). An exhausted CD8^+^ T cell signature, defined as the ratio of aggregated expressions of PD-1, LAG-3, and EOMES to the absolute CD8^+^ T cell fraction, was also elevated in tumors from Black compared to White patients (P<0.001), and associated with worse survival (10). Thus, while Black patients have higher TILs in the TME, such TILs are more likely to be in an exhausted state, which may explain patients’ poorer survival (10). There are also more tumor-associated macrophages (TAM), specifically M2 macrophages in tumors in Black patients (12) which may contribute to CD8^+^ T cell exhaustion (13, 14).CD8^+^ T cell exhaustion is a dynamic process where effector CD8^+^ T cells progressively lose their cytotoxic function and ability to produce effector cytokines. This process happens due to overstimulation, chronic tumor antigenic exposure, hypoxia, presence of regulatory T cells (Tregs) or myeloid-derived suppressor cells (MDSCs), and immunosuppressive cytokines [interleukin-10 (IL-10) and transforming growth factor-β (TGF-β)] (15, 16). Exhausted CD8^+^ T cells may express inhibitory receptors, such as, cytotoxic T lymphocyte antigen-4 (CTLA-4), programmed cell death protein 1 (PD-1), lymphocyte activation gene 3 protein (LAG-3), B and T-lymphocyte attenuator (BTLA), and T cell immunoglobulin domain and mucin domain protein 3 (TIM-3) on their surface. Exhausted cells have limited anti-tumor potential. To counteract this exhausted state, strategies using single or combined checkpoint blockade such as anti-CTLA-4, anti-PD-1/PDL-1, or LAG-3 blockade combined with anti-PD-1, or combination of anti-TIM3 with anti-PD-1 have proven effective in reversing CD8^+^ T cell exhaustion and restoring its cytotoxic function through epigenetic reprogramming (17) (**Figure 1**).

### Differences in cytokine and chemokine milieu across racial groups

Black patients with TNBC have higher plasma levels of IL-6, IL-8, and granulocyte colony-stimulating factor (12). Additionally, Black patients with breast cancer have an overall immunosuppressive TME due to the predominance of cytokines such as: IL-10 and TGF- β (18), and chemokines, such as, CCL7, CXCL8, CCL8, CCL17, CCL29, CCL25 (19) which may exert pro-tumor effects. Furthermore, tumors from Black women exhibit higher expression of Tregs (5) due to elevated level of CXCL12, which attracts pro-tumorigenic Tregs (18).

### Differences in genetic expressions across racial groups

Studies have shown that the mutation rates and intra-tumoral heterogeneity are higher in Black patients with TNBC compared to whites (20, 21). In a study by Kennan et al, Black patients were noted to have higher incidence of TP53 mutations compared to whites (20). Chang CS et al. reported that Black and white patients with TNBC had similar frequency of mutations in the commonly mutated genes such as TP53, MUC4, and MUC6 (21); however, Black patients are less likely to harbor PIK3CA and BRCA alterations compared to white patients, making agents targeting these mutations to be less beneficial in these patients (22). Black patients also have a higher number of mutations in JAK/STAT5, HER2, and ERBB2/ ERBB3 signaling pathways compared to whites, conferring poor prognosis (21). Moreover, they have increased expressions of vascular endothelial growth factor activator genes, leading to high tumor vascularization, which promotes tumor growth and metastasis (23). There is emerging evidence that Black patients with TNBC have high Enhancer of Zeste homologue 2 (EZH2) overexpression, which has been shown to induce AKT-dependent genomic instability in the TME and block BRCA1 function (24). In addition, significant enrichment of dysregulated genes associated with the WNT-ß catenin pathway was observed in Black patients, suggesting that activation of this pathway could contribute to the aggressive nature of disease seen in Black women (24). Interestingly, younger Black patients (<50 years) with TNBC were found to have a distinct DNA methylation landscape with significant differences in the expression of hormone pathway mediators such as ESR1, AR, and ESRRA, compared to white patients and older Black patients, suggesting a hormone unresponsive phenotype that may be associated with aggressive disease in this patient group (25). The key differences in tumor intrinsic and TME parameters between Black and White patients are summarized in **Table 1**.

**Table 1:** Characteristics of blood, tumor microenvironment and mutations in breast cancer among Black women.

Category	Key proteins and cell type	Expression pattern in Black vs. white patients	Effects	Reference
Serum	IL-6, IL-8, GCSF	High	Pro-inflammatory	(12)
	Resistin	High	Pro-inflammatory	(12)
	VEGF	High	Increased angiogenesis and tumor vascularization, promoting tumor growth and metastasis	(23)
TME	Exhausted CD8^+^ T cells	High	Loss of cytotoxic function and inability to produce effector cytokines	(10)
	Tumor-associated macrophages: M2 macrophages	High	CD8 T cell exhaustion	(12)
	IL-10 and TGF- β	High	Immune suppressive cytokines; pro-tumorigenic	(18)
	Chemokines: CCL7, CXCL8, CCL8, CCL17, CCL29, CCL25	High	Recruitment of tumor associated macrophages (M2 macrophages) to TME, promoting tumor progression and metastasis	(19)
Mutations (Germline/Somatic)	EZH2	Over expression	Induce genomic instability and block BRCA 1 function	(24)
	*JAK/STAT5, HER2, ERBB2/ERBB3* signaling pathways gene mutation	High	Dysregulated cell proliferation, differentiation, and survival	(21)
	*TP53*	High	Loss of tumor suppressor activity, uncontrolled cell proliferation	(20)
	*PIK3CA, BRCA* alterations	Low	Uncontrolled cell proliferation^+^	(22)

TME, tumor microenvironment; IL-6, interleukin-6; IL-8, interleukin-8; GCSF, granulocyte colony stimulating factor; IL-10, interlukein-10; TGF-β, Transforming growth factor beta; EZH2, Enhancer of Zeste homologue 2; VEGF, vascular endothelial growth factor. ^+^ Low incidence of PIK3CA, BRCA alterations makes targeted therapy less beneficial in black patients.

## Potential strategies to counteract CD8^+^ T cell exhaustion in TNBC among black patients

### Checkpoint inhibitors

Clinical trials that examined the impact of checkpoint inhibitors in TNBC in the neoadjuvant setting and assessed outcomes by race, are outlined in **Table 2**. In the NeoPACT (26) trial, which tested a combination of carboplatin, docetaxel and pembrolizumab, Black patients were observed to have higher stromal TILs than white patients. Black patients also achieved higher pathological complete response (pCR) rates compared to White patients (OR 3.27, 95% CI 1.01-10.64, P= 0.049) and longer 3-year event-free survival (EFS), supporting the notion of the potential benefit of immune checkpoint blockade in reversing CD8^+^ T cell exhaustion in Black patients. In contrast, findings from another trial by Foldi et al. (27) that included an extended cohort of Black patients in early-stage TNBC receiving neoadjuvant chemotherapy (doxorubicin-cyclophosphamide and paclitaxel) with durvalumab, showed no significant differences in pCR rate, EFS or overall survival by race. Additionally, there were no significant differences in stromal TILs or PDL-1 status by race. These varying outcomes by race in the two trials could be attributed to the use of different chemotherapeutic regimens and ICIs.

**Table 2:** Summary of clinical trials in TNBC with outcomes by race.

Clinical trial	Target	Treatment	Stromal TIL (median, %)		pCR (%)		3-year EFS	
			Black	White	Black	White	Black	White
Sharma et al. (26)	PD-1	Carboplatin, docetaxel and pembrolizumab	(40%)	(15%)	15/20 (79%)	49/93 (53%)	93%	81%
			*(P*=0.048)		(*P*= 0.049)		(*P*=0.40)	
Foldi et al. (27)	PDL-1	Doxorubicin, cyclophosphamide, paclitaxel and durvalumab	20%	10%	9/21 (43%)	22/47 (48%)	71.4%	78.3%
			*(P*=0.57)		(*P*=0.71)		(*P*=0.47)	

TNBC, triple negative breast cancer; PD-1, programmed cell death protein 1; PDL-1, programmed cell death ligand 1; TIL, tumor infiltrating lymphocytes; pCR, pathologic complete response; EFS, event free survival.

### Chemokine modulation

**Figure 2:**
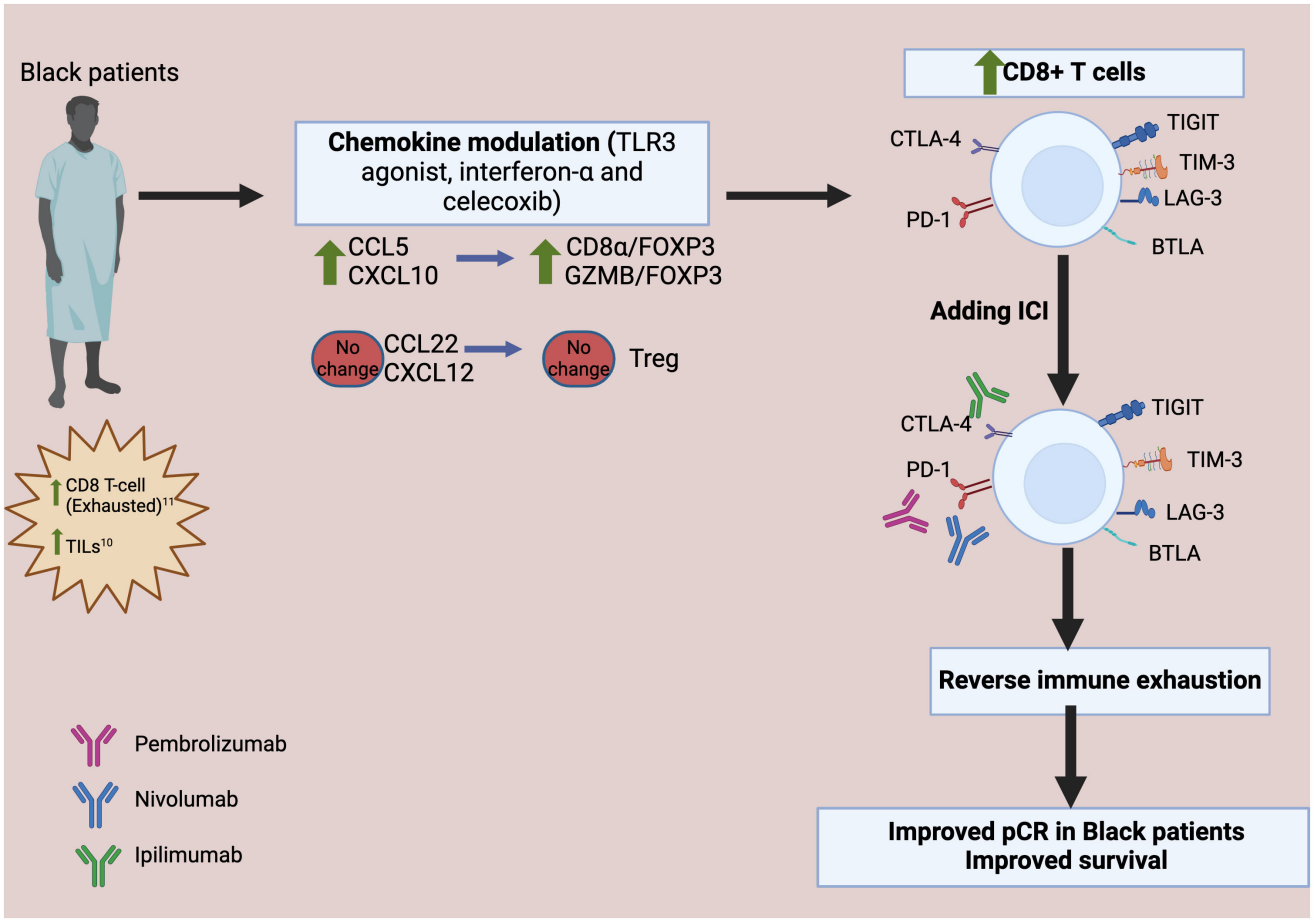
Proposed therapeutic interventions utilizing chemokine modulation and checkpoint blockade to reverse CD8^+^ T Cells exhaustion in Black Patients. The chemokine modulatory regimen selectively increases CCL5 and CXCL10, which attract cytotoxic T lymphocytes (CTL), while not increasing CCL22 or CXCL12. This leads to increased infiltration of the effector-type CTL, reflected by higher level of granzyme B FOXP3 is a marker of Treg; CD8α is a marker of CD8^+^ cytotoxic T cells. Upon the addition of immune checkpoint inhibitors (ICI), the exhaustion markers expressed in the exhausted CD8^+^T cells are blocked, thereby reversing immune exhaustion. TILs, tumor-infiltrating lymphocytes; GZMB, Granzyme B; CTL, cytotoxic T lymphocytes; Treg, regulatory T cells; pCR, pathologic complete response; CTLA-4, T lymphocyte antigen-4; PD-1, programmed cell death protein-1; LAG-3, lymphocyte activation gene 3 protein; BTLA, B and T-lymphocyte attenuator; TIM-3, T cell immunoglobulin domain and mucin domain protein 3; TIGIT, T cell immunoreceptor with Ig and ITIM domains.

Toll-like receptor 3 (TLR3) is an innate immune receptor expressed in immune cells and cancer cells that senses exogenous and endogenous endosomal double stranded RNA and is crucial in generating anti-microbial and tumor specific immune responses (28). Upon activation, it triggers a signal transduction pathway that leads to the production of pro-inflammatory cytokines, including type I interferon and chemokines, which recruits and activates immune cells (28). In addition to acting as an immunomodulator of TME, TLR3 can directly induce apoptosis of cancer cells (29). Studies have shown that interferon-α increases the surface expression of tumor-associated antigens in breast cancer cells and promote recognition of these antigens by T cells (30). Moreover, interferon-α promotes expression of molecules such as granzyme B, and perforin in effector T cells, which increases the cytotoxic effect of T cells (30). Our preclinical work demonstrated that combining toll-like receptor 3 (TLR3) ligands with interferon-α synergistically enhances the desirable CTL attractants and reduces the Treg and MDSC attractants in the TME (31, 32). In our recent clinical trial (NCT03599453) (33), the chemokine modulatory (CKM) regimen composed of a TLR3 agonist, interferon-α and celecoxib was administered to patients with metastatic TNBC, followed sequentially by pembrolizumab. CKM selectively increased intratumoral CTL attractants such as CCL5 or CXCL10, but not the Treg attractants CCL22 or CXCL12. Our trial further indicates an increased infiltration of CTLs into the tumor, and intratumoral granzyme B signature was also elevated. These observations point to the potential of CKM as a complementary strategy to immune checkpoint inhibitors, as CKM results in infiltration of CD8^+^T cells into the tumor and immune checkpoint inhibitors could reverse the exhaustion signature on these CD8^+^T cells. Both strategies would work synergistically to rejuvenate CD8^+^ T cell function (**Figure 2**).

## Future directions

Given the link between biased chemokine patterns in Black TNBC patients, compared to Caucasian patients observed in our recent studies and the ability of CKM to correct such bias observed in our recent Phase I study, we propose that a dual strategy combining chemokine modulation with immune checkpoint inhibitors may have the potential to emerge as a novel approach to address racial disparity within the TME of TNBC, especially among Black patients.

## Data availability statement

The original contributions presented in the study are included in the article/supplementary material. Further inquiries can be directed to the corresponding author.

## Author contributions

MA: Writing – original draft, Writing – review & editing. AR: Writing – review & editing, Conceptualization. JK: Writing – review & editing. PK: Writing – review & editing. SY: Writing – review & editing. SG: Conceptualization, Supervision, Writing – original draft, Writing – review & editing.
